# Cell Type-Specific Neuroprotective Activity of Untranslocated Prion Protein

**DOI:** 10.1371/journal.pone.0013725

**Published:** 2010-10-28

**Authors:** Elena Restelli, Luana Fioriti, Susanna Mantovani, Simona Airaghi, Gianluigi Forloni, Roberto Chiesa

**Affiliations:** 1 Dulbecco Telethon Institute, Milan, Italy; 2 Department of Neuroscience, Mario Negri Institute for Pharmacological Research, Milan, Italy; National Institutes of Health, United States of America

## Abstract

**Background:**

A key pathogenic role in prion diseases was proposed for a cytosolic form of the prion protein (PrP). However, it is not clear how cytosolic PrP localization influences neuronal viability, with either cytotoxic or anti-apoptotic effects reported in different studies. The cellular mechanism by which PrP is delivered to the cytosol of neurons is also debated, and either retrograde transport from the endoplasmic reticulum or inefficient translocation during biosynthesis has been proposed. We investigated cytosolic PrP biogenesis and effect on cell viability in primary neuronal cultures from different mouse brain regions.

**Principal Findings:**

Mild proteasome inhibition induced accumulation of an untranslocated form of cytosolic PrP in cortical and hippocampal cells, but not in cerebellar granules. A cyclopeptolide that interferes with the correct insertion of the PrP signal sequence into the translocon increased the amount of untranslocated PrP in cortical and hippocampal cells, and induced its synthesis in cerebellar neurons. Untranslocated PrP boosted the resistance of cortical and hippocampal neurons to apoptotic insults but had no effect on cerebellar cells.

**Significance:**

These results indicate cell type-dependent differences in the efficiency of PrP translocation, and argue that cytosolic PrP targeting might serve a physiological neuroprotective function.

## Introduction

The cellular prion protein (PrP^C^) is a glycosylphosphatidylinositol (GPI)-anchored cell-surface glycoprotein with unclear function that is expressed at the highest level by neurons in the central nervous system [1], [2], [3]. Conversion of PrP^C^ into an abnormal, misfolded isoform plays a key role in prion diseases, which are invariably fatal neurodegenerative disorders that can arise sporadically, be inherited due to mutations in the gene encoding PrP, or acquired through infection [4].

Research on prion diseases has focused on how perturbations of PrP^C^ biosynthesis and metabolism may trigger the neurodegenerative process [5]. PrP^C^ is co-translationally translocated into the rough endoplasmic reticulum (ER), where the N-terminal signal peptide (SP) is cleaved, and the GPI anchor is added concurrently with removal of a C-terminal signal sequence. In the ER, the PrP polypeptide undergoes oxidative folding with formation of a single disulphide bond, and the protein is variably modified at two N-glycosylation sites, resulting in a mixture of di-, mono- and unglycosylated forms [6]. After transit in the mid-Golgi, where the immature, core-glycosylated molecules are complex-glycosylated, PrP is transported through the later compartments of the secretory pathway and delivered to the cell surface, where it resides in lipid rafts [7].

The observation that pharmacological inhibition of the proteasome led to accumulation of an unglycosylated PrP species in neuroblastoma N2a cells [8], [9] was interpreted as evidence that part of the newly synthesized PrP was constitutively recognized as misfolded by the ER quality control and diverted to the ER-associated degradation (ERAD) pathway, which implies retrograde transport from the ER lumen to the cytosol, deglycosylation by cytosolic N-glycanases, and proteasomal degradation [10]. Conditions favoring PrP misfolding such as germline or somatic mutations, and/or reduced proteasome function, might therefore lead to accumulation of potentially neurotoxic cytosolic PrP. Consistent with the idea that ERAD-diverted PrP could be neurotoxic if not properly degraded, forced expression of PrP in the cytosol caused degeneration of cerebellar granule neurons, and anatomical and functional abnormalities in the forebrain of transgenic (Tg) mice [11], [12], [13].

Cytosolic PrP could also be generated by an ERAD-independent mechanism. During PrP biosynthesis a subset of molecules failed to translocate into the ER lumen and ended up in the cytosol [14], [15], because of an intrinsic inefficiency of the PrP signal sequence [16]. The amount of untranslocated PrP increased during ER stress [17], [18], providing an alternative mechanism for generating potentially neurotoxic cytosolic PrP [19].

However, several observations undermine the idea that cytosolic PrP is invariably neurotoxic. In non-pathogenic conditions, PrP was found in the cytoplasm of some neuronal populations in the hippocampus, neocortex and thalamus, with no signs of neurodegeneration [20], [21], [22]. Then too, analysis of cytosolic PrP activity in different cells produced conflicting results: whereas some studies confirmed the toxicity [11], [16], [23], [24], [25], others did not [15], [26], [27], and some brought to light a protective effect against Bax-mediated cell death [28], [29]. These observations raised the possibility that cells of different neural origin could differ in their propensity to synthesize PrP in the cytosol, and that this isoform could have cell type-specific biological activities.

To explore this, we investigated cytosolic PrP biogenesis and effects on cell survival in primary neuronal cultures from different mouse brain regions. Here we show that when the proteasome is inhibited, an unglycosylated form of PrP accumulates in cortical and hippocampal cells, but not in cerebellar granule neurons (CGN). This form contains uncleaved signal peptides, indicating that it corresponds to PrP molecules that have escaped translocation into the ER. Consistent with this, an inhibitor of protein translocation increased the amount of cytosolic PrP in cortical and hippocampal neurons, and induced its synthesis in CGNs. Untranslocated PrP was associated with an increase in the resistance of cortical and hippocampal cells to apoptosis, but had no such effect on cerebellar granules. These findings support the conclusion that cytosolic PrP is not neurotoxic, and suggest that selective targeting of nascent PrP to the cytosol might fulfill a neuroprotective function.

## Results

### Untranslocated PrP is Detected in Cultured Cortical and Hippocampal Neurons, but not in Cerebellar Granules

**Figure 1:**
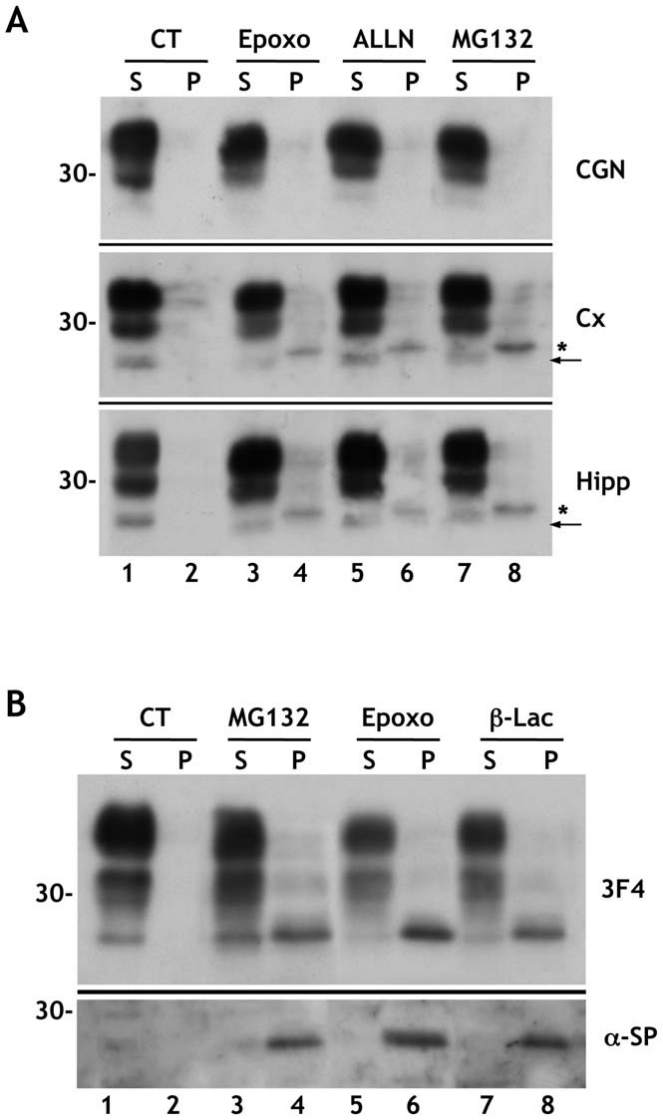
Proteasome inhibitors induce accumulation of insoluble, untranslocated PrP in primary neurons. (A) Cerebellar granule neurons (CGN), cortical (Cx) and hippocampal (Hipp) neurons from C57BL/6J mice were treated for 24h with 5 μM epoxomicin (Epoxo), 100 μM ALLN, 5 μM MG132, or the vehicle alone (CT). Cell lysates were centrifuged at 186,000 x *g* for 40 min, and PrP in the supernatants (S) and pellets (P) was visualized by immunoblotting with antibody P45-66. The asterisks and arrows indicate bands corresponding to untranslocated PrP and mature, unglycosylated PrP, respectively. (B) Cortical neurons from Tg(WT-E1) mice were exposed to 5 μM MG132, epoxomicin (Epoxo), lactacystin β-lactone (β-Lac) or the vehicle alone (CT). After 24 hours, cells were lysed and centrifuged at 186,000 x *g* for 40 min. PrP was visualized by immunoblotting with antibody 3F4 (upper panel), and with an antibody against the N-terminal signal peptide (α-SP) (lower panel). Molecular mass markers are in kilodaltons.

We investigated whether cytosolic PrP was detectable in primary neurons cultured from the neocortex, hippocampus and cerebellum of newborn mice. Because cytosolic PrP is rapidly degraded by the proteasomes [14], [15], cells were treated with a panel of different proteasome inhibitors. Lactacystin-β-lactone, MG132 (Z-Leu-Leu-Leu-al), ALLN (Ac-Leu-Leu-NorLeu-al) or epoxomycin caused accumulation of an insoluble form of PrP of approximately 27 kDa in cortical and hippocampal neurons (Fig. 1A, middle and bottom panels, and Fig. 1B top panel). This form had a larger molecular mass then mature, unglycosylated PrP in the soluble fractions, and was recognized by an antibody (α-SP), which selectively reacts with the N-terminal signal peptide of PrP [30] (Fig. 1B, lower panel), indicating that it corresponded to the untranslocated form of cytosolic PrP previously described in transfected cells (hereafter referred to as SP-PrP) [14], [15], [16], [17]. In cortical and hippocampal cells SP-PrP was first detected after 2 h treatment, and reached a maximum within 8 h (data not shown) which, based on quantitative evaluation of Western blots, corresponded to approximately 10% of total PrP. Consistent with previous findings [14], [15], SP-PrP was not detected in proteasome inhibitor-treated CGN (Fig. 1A, top panel). PrP levels were similar in the different neuronal cultures, ruling out that the failure to detect SP-PrP in CGN was due to lower PrP expression.

**Figure 2:**
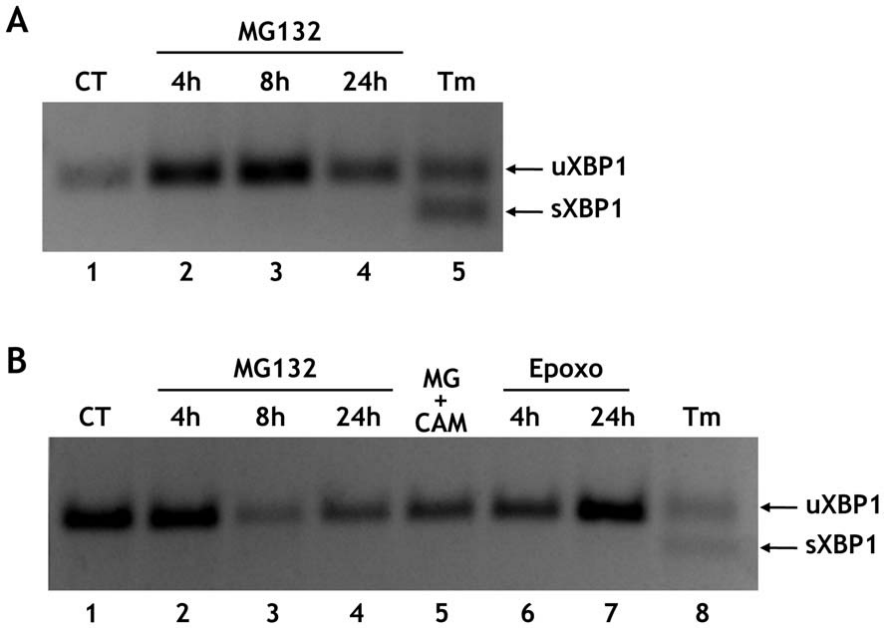
Proteasome inhibitors do not induce ER stress in primary neurons. Cortical (A) or cerebellar granule neurons (B) were treated with 5 μM MG132 or epoxomicin (Epoxo) for the times indicated, with 5 μM MG132 and 10 μM CAM741 for 18 h, or 5 μg/ml tunicamycin (Tm) for 8 h. After treatment, total RNA was extracted and analyzed by reverse transcription-PCR. XBP1 splicing was determined by the appearance of rapidly migrating spliced XBP1 in tunicamycin-treated cells. The arrows point to unspliced (uXBP1) and spliced (sXBP1) transcripts.

During acute ER stress, PrP is prevented from translocating into the ER and is routed to the cytosol [17], [18]. To see whether proteasome inhibitors activated ER stress pathways in neurons, we followed the splicing of X-box binding protein 1 (XBP1) mRNA transcripts [31], [32]. Splicing was readily detected in cells treated with tunicamycin, which inhibits protein glycosylation and induces ER stress by perturbing the folding efficiency in the ER (Fig. 2A, lane 5, and 2B, lane 8). No XBP1 splicing was observed in proteasome inhibitor-treated cells (Fig. 2). There was also no increase in the ER stress-regulated protein Grp78/Bip in cells producing untranslocated PrP (data not shown). Thus, accumulation of SP-PrP was due not to an indirect effect of ER stress on PrP translocation, but to impaired degradation of a cytosolic pool of native PrP molecules.

**Figure 3:**
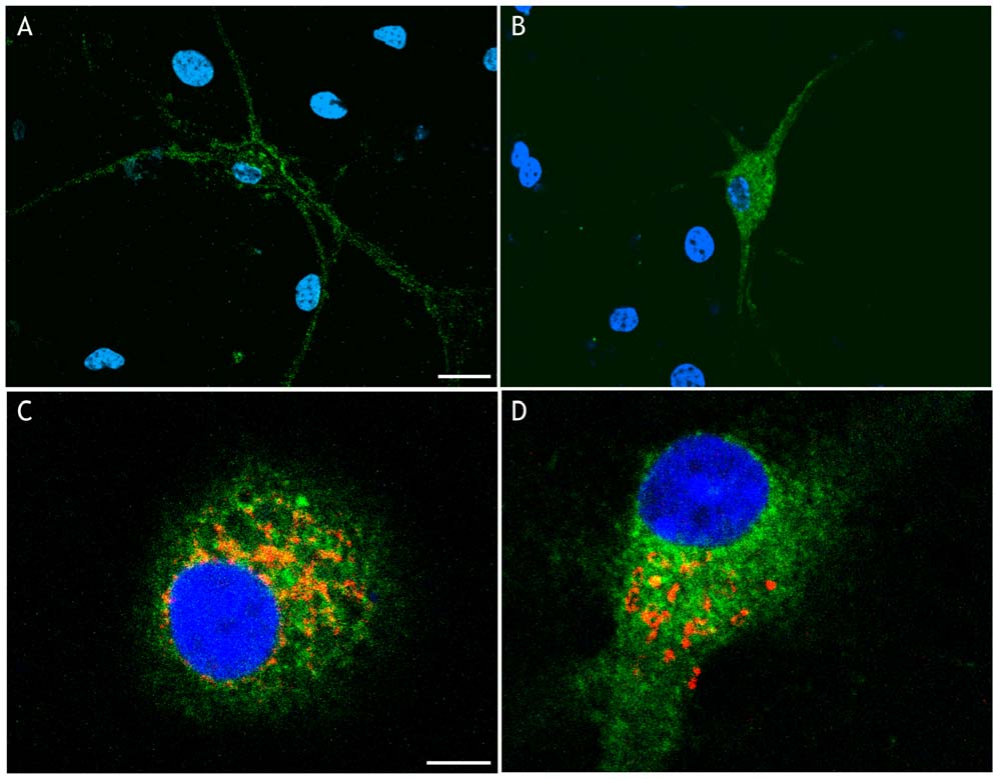
Untranslocated PrP shows cytosolic localization. Hippocampal neurons from C57BL/6J mice were transfected with a plasmid encoding PrP-EGFP (green). Twelve days after transfection cells were exposed to the vehicle (A) or treated with 10 μM CAM741 for 24 h plus 5 μM MG132 during the last 6 h (B–D). Cells were then fixed and reacted with DAPI (blue) to stain the nuclei. Cells in C and D were also immunostained with an anti-PDI or anti-golgin antibody (red) to visualize the ER and Golgi, respectively. Scale bar  = 10 μm in A (also applicable to B), and 5 μm in C (also applicable to D).

To determine the cellular localization of SP-PrP, we transfected hippocampal neurons with a plasmid encoding a PrP-enhanced green fluorescent protein (PrP-EGFP) fusion molecule [33], and induced robust synthesis of untranslocated PrP-EGFP by treating the cells with an inhibitor of PrP translocation (see below). We imaged PrP-EGFP in fixed, DAPI-stained cells by confocal microscopy to visualize its localization in relation to the nucleus. Consistent with previous immunolocalization of a non-fluorescent version of PrP in cultured neurons [15], PrP-EGFP distributed on the cell soma and along the neurites of untreated cells (Fig. 3A). There was also a fraction in intracellular compartments that co-localized with the ER and Golgi (not shown), as expected for proteins in transit towards the cell surface. In treated neurons, PrP-EGFP showed a fine punctate cytoplasmic fluorescence (Fig. 3B–D), the majority of which did not co-localize with ER or Golgi markers (Fig. 3C and D, respectively).

### Neurons Synthesizing SP-PrP Have Enhanced Resistance to Proteasome Inhibitor- and Staurosporine-induced Cell Death

**Figure 4:**
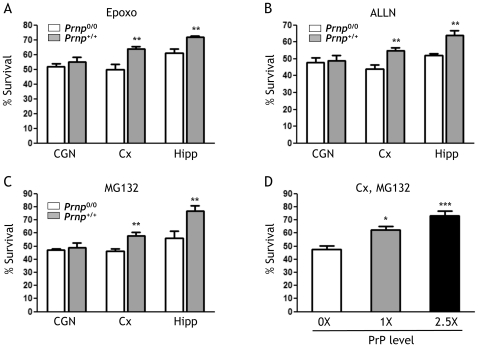
Neurons synthesizing SP-PrP are more resistant to proteasome inhibitors. Cerebellar granule neurons (CGN), cortical (Cx) and hippocampal (Hipp) neurons prepared from C57BL/6J (*Prnp* ^+/+^) and PrP knockout (*Prnp* ^0/0^) mice were exposed to 1 μM epoxomicin (A), 100 μM ALLN (B) or 5 μM MG132 (C). Cell survival was quantified after 24 h by MTT assay and expressed as a percentage of the values for cells treated with the vehicle. Data are the mean ± SEM of 12–24 replicates from 2–4 independent experiments; **p<0.01 by Tukey-Kramer test. (D) Cortical neurons were prepared from *Prnp* ^0/0^ (PrP level: 0X), *Prnp* ^+/+^ (PrP level: 1X), and Tg(WT-E1)^+/−^/*Prnp* ^+/0^ (PrP level: ∼2.5X) littermates obtained by crossing Tg(WT-E1)^+/−^/*Prnp* ^+/0^ and *Prnp* ^+/0^ mice. Cell viability was analyzed after 24-h treatment with 5 μM MG132 and expressed as a percentage of the values for cells treated with the vehicle. Data are the mean ± SEM of 8-23 replicates from 2 independent experiments; *p<0.05, ***p<0.001 by Dunnett's test.

To test the effect of SP-PrP on the viability of cultured neurons we used a previously described experimental paradigm [11], [15]. Neurons cultured from PrP knockout (*Prnp* ^0/0^) and wild-type (*Prnp* ^+/+^) mice were exposed to proteasome inhibitors and their viability was evaluated after 24 h. There was no difference in viability between *Prnp* ^0/0^ and *Prnp* ^+/+^ CGN; in contrast, cortical and hippocampal neurons from *Prnp* ^+/+^ mice were significantly more resistant to the inhibitors than their *Prnp* ^0/0^ counterparts (Fig. 4A–C). Supraphysiological PrP expression further increased cortical cell resistance to the inhibitors, indicating a dose-dependent effect of PrP expression on neuronal survival (Fig. 4D). The fact that the cells that survived best were those synthesizing SP-PrP suggested that this isoform could have cytoprotective activity.

**Figure 5:**
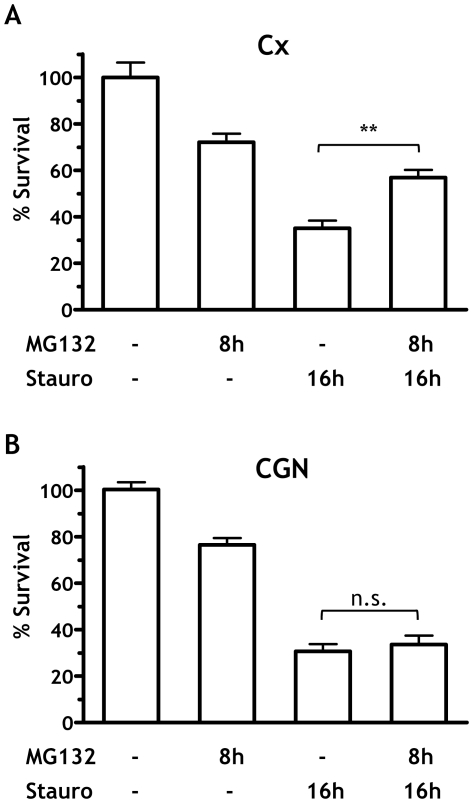
MG132 protects cortical but not cerebellar granule neurons from staurosporine-induced cell death. Cortical (A, Cx) and cerebellar granule neurons (B, CGN) from C57BL/6J mice were exposed to 5 μM MG132 for 8 h to induce accumulation of SP-PrP in cortical neurons, and treated with 100 nM staurosporine or not for 16 h. Cell survival was quantified at the end of the treatment (24 h) by MTT assay and expressed as a percentage of the values for cells treated with the vehicle. Data are the mean ± SEM of 20–25 replicates from four independent experiments; **p<0.01 by Bonferroni test.

To test this, we investigated whether SP-PrP protected neurons from the toxic effect of staurosporine, a prototypic inducer of apoptosis [34], [35]. Cortical and cerebellar neurons were treated with or without MG132 for 8 hours (long enough to induce accumulation of SP-PrP in cortical cells), then incubated for 16 hours with or without staurosporine, before evaluating cell viability by MTT assay. Statistical analysis showed a significant protective effect of MG132 against staurosporine-induced cell death in cortical neurons (Fig. 5A), but no effect on CGN viability (Fig. 5B). Thus SP-PrP had an anti-apoptotic effect in cortical cells, consistent with the protective effect of cytosolic PrP against Bax-induced cell death [28], [29].

### Inhibition of Protein Translocation Induces Accumulation of SP-PrP in CGN

**Figure 6:**
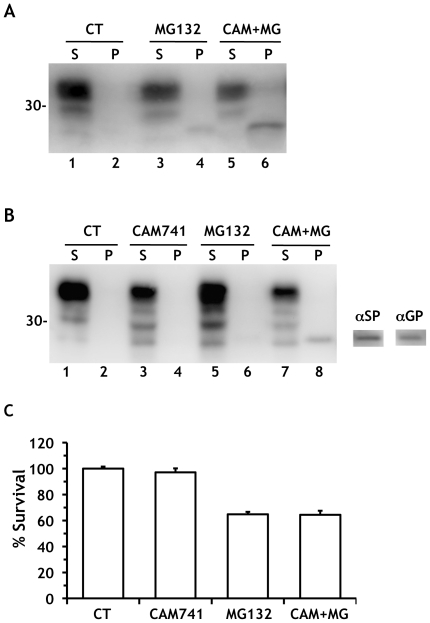
SP-PrP has no effect on viability of cerebellar granule neurons. (A) Hippocampal neurons from C57BL/6J mice were treated with 5 μM MG132 alone or with 10 μM CAM741 for 18 h, and PrP was analyzed by Western blot as described in the legend to Fig. 1. Note the higher level of SP-PrP in the presence of CAM741 (compare lanes 4 and 6). (B) Cerebellar granule neurons from C57BL/6J mice were treated with 10 μM CAM741, 10 μM MG132 or with the two drugs simultaneously for 24 h, before Western blot analysis with antibody 12B2. The PrP band in lane 8 also reacted with the α-SP and α-GP antibodies (on the right). (C) Cell survival was quantified by MTT assay and expressed as a percentage of the values for cells treated with the vehicle. Data are the mean ± SEM of 18–20 replicates from two independent experiments. The Bonferroni test did not find any difference between MG132 and CAM+MG groups.

Transgenic mice engineered to express PrP in the cytosol show massive degeneration of CGN, suggesting that cytosolic PrP may be selectively toxic to these cells [11]. To test this we sought ways to induce cytosolic PrP localization in cultured CGN. CAM741, a cyclopeptolide that inhibits co-translational translocation by interfering with the correct insertion of the signal peptide into the translocon [36], [37], increased the amount of SP-PrP in hippocampal and cortical neurons (Fig. 6A, compare lanes 4 and 6, and data not shown). CAM741 induced an unglycosylated PrP species in CGN (Fig. 6B, lanes 7 and 8), which was confirmed identical to SP-PrP by reactivity with the α-SP antibody and an antibody directed against the C-terminal GPI-anchoring signal (α-GP) [17] (Fig. 6B, right). When we analyzed how SP-PrP accumulation affected CGN viability, we found no significant difference between cells treated with MG132 alone or in combination with CAM741 (Fig. 6C), even though the latter accumulated SP-PrP (Fig. 6B, lanes 7 and 8).

**Figure 7:**
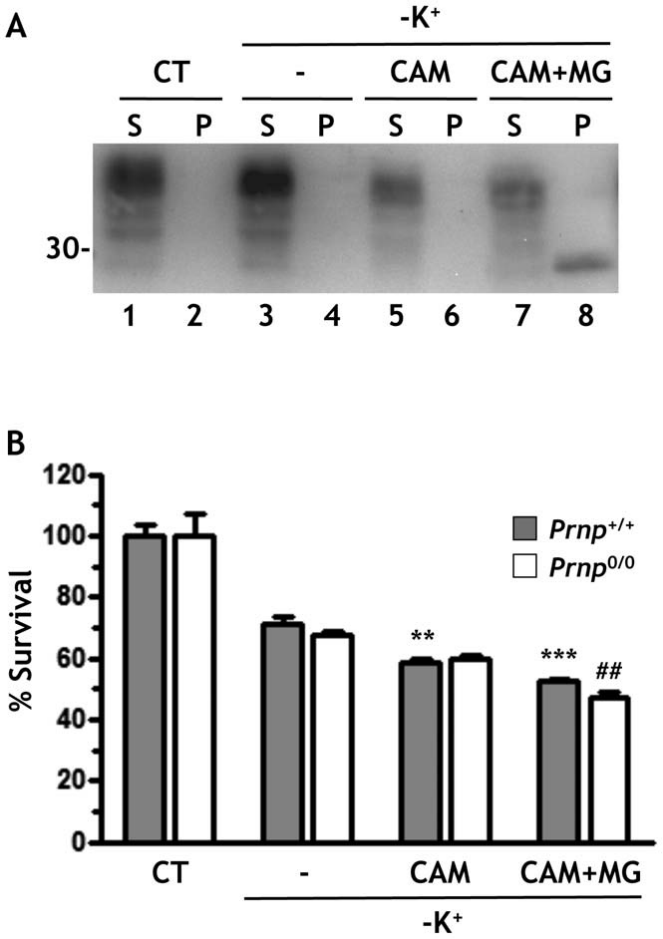
SP-PrP does not affect the cerebellar granule neurons' response to serum and potassium deprivation. (A) Cerebellar granule neurons from C57BL/6J mice were shifted to serum-free medium containing 5 mM KCl, and left untreated (-) or treated with 10 μM CAM741 alone or in combination with 10 μM MG132 to induce accumulation of SP-PrP. After 24 h cells were analyzed by Western blot with antibody 12B2 to verify the induction of SP-PrP. (B), Cell survival was quantified by MTT assay and expressed as a percentage of the values for untreated cells. Data are the mean ± SEM of 12 replicates; **p<0.01, ***p<0.001 vs -K^+^ in *Prnp* ^+/+^; ^##^ *p*<0.01 vs -K^+^ in *Prnp* ^0/0^ by Bonferroni test.

Next, we tested the effect of SP-PrP in CGN deprived of serum and potassium, a condition that induces apoptotic cell death [38]. After 24 h of deprivation, CGN viability was reduced by ∼30% (Fig. 7B). CAM741 alone, or combined with MG132 to induce accumulation of SP-PrP (Fig. 7A, lanes 7 and 8), caused a small but significant decrease of cell survival (Fig. 7B, gray bars). However, the same happened in CGN from *Prnp* ^0/0^ mice (Fig. 7B, white bars), indicating that the loss of cell viability was due to a toxic effect of the treatment, independent of SP-PrP.

Finally, we used an experimental paradigm similar to that shown in Fig. 5 to test whether CAM741-induced SP-PrP protected CGN from staurosporine toxicity. SP-PrP had no effect on cell viability (not shown). Thus, in CGN, SP-PrP was not toxic, and did not protect from apoptosis.

## Discussion

The cellular pathways by which cytosolic PrP is generated and the biological activity of this species have been debated. Previous investigations have used transfected cells or transgenic mice engineered to express artificial PrP molecules in the cytosol, making extrapolation to physiological conditions difficult. In the present study we investigated the biogenesis and biological activity of cytosolic PrP produced endogenously in primary neurons from different mouse brain regions. The efficiency of PrP compartmentalization in the secretory pathway varied significantly for different neurons, with cortical and hippocampal cells synthesizing an untranslocated form of cytosolic PrP, which was not present in cerebellar granules. Synthesis of untranslocated PrP caused no toxicity to neurons –in fact, it increased resistance to apoptosis. These data indicate that inefficient co-translational translocation during biogenesis is the primary source of cytosolic PrP in neurons, and raise the possibility that cytosolic targeting of nascent PrP molecules might be physiologically regulated for cellular benefit.

Proteasome inhibitors induced accumulation of an unglycosylated form of PrP in primary neurons. This form had a higher molecular mass than mature, unglycosylated PrP and carried the N- and C-terminal signals that are cleaved in the ER lumen. Thus, cytosolic PrP represents untranslocated molecules that have never entered the ER rather than retrogradely translocated PrP, which would lack both signal peptides. In cultured neurons untranslocated PrP showed a fine punctate cytosolic localization reminiscent of that in certain brain neurons [20], [21], [22].

ER stress favors accumulation of untranslocated PrP by activating a “preemptive” quality control mechanism that inhibits protein translocation [17], [18]. Therefore it was important to verify whether proteasome inhibitors activated ER stress pathways in neuronal cells [39]. There was no evidence of splicing of the mRNA encoding XBP-1 or increases in the levels of the ER stress-regulated protein Grp78/BiP. This indicated that SP-PrP accumulation in response to proteasome inhibition was due not to an indirect effect of ER stress, but to a pool of short-lived PrP molecules in the cytosol.

Unlike cortical and hippocampal cells, we detected no SP-PrP in cerebellar granules unless translocation was pharmacologically inhibited. This is in line with analyses of the mouse brain, where cytosolic PrP was detected in neurons of the neocortex and hippocampus, but not the cerebellum [20], [21], and suggests that cell-specific factors influence PrP translocation. Consistent with this conclusion, the efficiency of PrP compartmentalization in the secretory pathway varied markedly in different cell lines [40].

The molecular steps leading to signal-mediated protein segregation into the mammalian ER include recognition of the nascent polypeptide chain by the signal recognition particle (SRP), followed by SRP-dependent targeting to the ER membrane and transfer to the Sec61 translocon. The nascent polypeptide is then inserted into the protein-conducting channel of the translocon, allowing protein translocation concurrently with its synthesis [41]. The fact that the amount of SP-PrP increased in response to CAM741, which interferes with the correct insertion of the signal peptide into the translocon [36], [37], suggests that a post-targeting interaction between the signal and the Sec61 channel is primarily involved in PrP translocation, a conclusion also emerging from other studies [18], [42].

Several “accessory” components influence post-targeting PrP translocation, such as the translocon-associated protein complex TRAP [43], and chaperones and disulfide isomerases that associate with the cytoplasmic side of Sec61 [44]. It remains to be established whether regulated expression of these proteins governs SP-PrP synthesis in neuronal cells.

Consistent with evidence of an anti-apoptotic function of cytosolic PrP [28], SP-PrP protected neurons against the toxicity of proteasome inhibitors and staurosporine, which activate the intrinsic apoptotic pathway [35], [45]. This effect was seen in cortical and hippocampal cells, but not in cerebellar granules where CAM741 induced SP-PrP. This suggests there are specific factors that influence the activity of SP-PrP, for example PrP-interacting proteins that may be selectively expressed or functionally more critical in some cell types than others. A number of proteins have been identified that could interact with PrP in the cytosol and mediate neuroprotection, including the anti-apoptotic protein Bcl-2 [46] and the neurotrophin receptor-interacting MAGE homologue, NRAGE [47]. However, preliminary attempts to identify a physical interaction between SP-PrP and these candidate proteins have not been rewarding (unpublished data). The fact that untranslocated PrP has a short half-life [14] suggests it may engage only in transient interactions.

The neuroprotective activity of untranslocated PrP produced endogenously in cultured neurons contrasts with the neurotoxicity of forced cytosolic PrP expression by transgenesis. A PrP construct lacking N- and C-terminal signal peptides, designed to mimic cytosolic PrP from retrotranslocation, caused massive degeneration of CGN in transgenic mice [11]. Although this observation is provocative, the lack of evidence of cytosolic PrP generation by retrotranslocation in neuronal cells [14,15, and this study] raises questions about its pathologic importance.

Rane and colleagues generated Tg mice expressing a variant of hamster PrP carrying the interferon-γ signal peptide (Ifn-PrP), which was constitutively translocated at low levels [19]. Transgenic mice expressing small amounts of Ifn-PrP (1/6th of the endogenous PrP mRNA level) were smaller than non-Tg littermates and developed mild ataxia with modest spongiform changes at 18–24 months of age. Tg lines with higher Inf-PrP expression could not be established because of embryonic and neonatal lethality [19]. This indicates that constitutive expression of untranslocated PrP during development is highly detrimental, perhaps underscoring the importance of regulated PrP translocation early in the mouse's life.

The signal sequence of PrP has evolved to maintain a slight but measurable inefficiency in interaction with the translocon [42], [48]. This allows generation of multiple topological forms of the protein, including untranslocated PrP and ^Ctm^PrP, a transmembrane variant with neurotoxic properties [49], [50], [51]. It is therefore tempting to speculate that PrP might have acquired the ability to adopt cytosolic or transmembrane topologies with opposite effects on neuronal survival for a functional purpose, perhaps to fine-tune signaling cascades that control cell fate in the developing brain [2], [52]. In certain species cytosolic PrP can be generated by alternative initiation of translation, which produces PrP molecules with short signal peptides unable to negotiate entry into the ER [53], [54]. Thus different molecular strategies might have evolved to produce untranslocated PrP.

A number of secretory and membrane proteins appear to have inefficient signal sequences for beneficial functions. Thus, during ER stress, proteins such as PrP are prevented from entering the secretory pathway to alleviate the burden of the folding and secretory transport machineries of the cell [17], [18]. We have provided evidence that a pool of untranslocated cytosolic PrP is synthesized in certain neurons in the absence of ER stress, indicating the existence of cell type-specific pathways that control PrP translocation in physiological conditions. We also report a beneficial effect of untranslocated PrP on neuronal survival, suggesting a new teleological argument for the evolutionary conserved inefficiency of the PrP signal sequence. The challenge for future studies will be to elucidate the cellular mechanisms regulating the biogenesis of untranslocated PrP, and the downstream molecular pathways that mediate its biological effect.

## Materials and Methods

### Mice

Production of Tg(WT-E1) mice overexpressing mouse wild-type PrP (moPrP) tagged with an epitope for the monoclonal antibody 3F4 has been reported elsewhere [55]. *Prnp* ^0/0^ mice [56] with a pure C57BL/6J background were obtained from the European Mouse Mutant Archive (Monterotondo, Rome, Italy). C57BL/6J mice were purchased from Charles River Laboratories.

### Ethics Statement

All procedures involving animals were conducted according to European Union (EEC Council Directive 86/609, OJ L 358,1; December 12, 1987) and Italian (D.L. n.116, G.U. suppl. 40, February 18, 1992) laws and policies, and were in accordance with the United States Department of Agriculture Animal Welfare Act and the National Institutes of Health Policy on Humane Care and Use of Laboratory Animals. They were reviewed and approved by the Mario Negri Institute Animal Care and Use Committee that includes *ad hoc* members for ethical issues (ID 9/1/01). Animal facilities meet international standards and are regularly checked by a certified veterinarian who is responsible for health monitoring, animal welfare supervision, experimental protocols and review of procedures.

### Cell Culture

Primary neuronal cultures were prepared as previously described [15], [57]. Briefly, cerebella were dissected, sliced into ∼1-mm pieces and incubated in Hank's balanced salt solution (HBSS, Gibco) containing 0.3 mg/ml trypsin (Sigma) at 37°C for 15 min. Trypsin inhibitor (Sigma) was added to a final concentration of 0.5 mg/ml and the tissue was mechanically dissociated by passing through a flame-polished Pasteur pipette. Cells were plated at 350–400,000 cells/cm^2^ on poly-L-lysine (0.1 mg/ml)-coated plates. Cells were maintained in Basal Medium Eagle (Gibco) supplemented with 10% dialyzed fetal bovine serum (FBS, Sigma), penicillin/streptomycin and KCl 25 mM, at 37°C in an atmosphere of 5% CO_2_, 95% air.

Cortical and hippocampal neurons were prepared from two-day-old animals as described [57]. Brain tissue was sliced into ∼1-mm pieces and incubated in HBSS (Gibco) containing 20 U/ml papain (Sigma) at 34°C for 30 min. Trypsin inhibitor (Sigma) was added to a final concentration of 0.5 mg/ml and the tissue was mechanically dissociated by passing through a flame-polished Pasteur pipette. Cells were plated at 150–250,000 cells/cm^2^ on poly-D-lysine-coated (25 μg/ml) plates and maintained in Neurobasal Basal Medium (Gibco) supplemented with B27 (Gibco), penicillin/streptomycin and glutamine 2 mM. To reduce the number of non-neuronal cells, aphidicolin (3.3 μg/ml, Sigma) was added to the medium 48 h after plating. Non-neuronal contamination was less than 3%.

Cell viability was assessed by measuring the cellular reduction of 3-(4,5-dimethylthiazol-2-yl)-2,5-diphenyl tetrazolium bromide (MTT) to formazan [57]. Cells were incubated for 3 h at 37°C with 0.4 mg/ml MTT, dissolved in 0.04N HCl in 2-propanol, and analyzed spectrophotometrically at 540 nm with an automatic microplate reader (Labsystems Multiskan MS).

### Cell Transfection

Hippocampal neurons were transfected with a plasmid encoding a moPrP molecule containing a monomerized version of enhanced green fluorescent protein (EGFP) inserted after codon 34 [33], using the Nucleofector device and the Mouse Neuron Nucleofector Kit (Lonza). Cells were then pelleted, resuspended in RPMI 1640 (Gibco) containing 10% FBS (Sigma) and 2 mM glutamine, and plated at 300,000 cells/cm^2^ on poly-D-lysine (25 μg/ml)-coated plates. After 2 h the medium was replaced with Neurobasal Basal Medium (Gibco) supplemented with B27 (Gibco), penicillin/streptomycin and glutamine 2 mM, and cells were maintained at 37°C in an atmosphere of 5% CO_2_, 95% air. They were analyzed after 12–14 days in culture.

### Antibodies

Monoclonal antibody 3F4 was diluted 1:5,000 [58]. Rabbit polyclonal antibody P45-66, raised against a synthetic peptide encompassing residues 45–66 of moPrP, was used at 1∶2,500 [59]. An affinity-purified polyclonal rabbit antibody (α-SP) that selectively recognizes forms of moPrP containing an uncleaved signal peptide was used at 1∶150 [30]. A rabbit polyclonal antibody (α-GP) raised against the C-terminal sequence of moPrP, which is removed before the addition of the GPI anchor was used at 1∶1,000 [17]. Monoclonal antibody 12B2 against moPrP sequence 88–92 was used at 1∶5,000 [60]. Polyclonal rabbit antibodies against protein disulfide isomerase (PDI, Sigma) and giantin (Covance) were used at 1∶500.

### Biochemical Analysis

To assay detergent insolubility, cells were lysed in 10 mM Tris pH 7.5, 100 mM NaCl, 0.5% sodium deoxycholate and 0.5% Nonidet P-40 containing protease inhibitors (pepstatin and leupeptin, 1 μg/ml; phenylmethylsulfonyl fluoride, 0.5 mM; and ethylenediaminetetraacetic acid, 2 mM). Lysates corresponding to 300 μg of protein were centrifuged at 186,000 x *g* for 40 min in a Beckman Optima Max-E ultracentrifuge. Proteins in the pellet and supernatant were separated by sodium dodecyl sulphate-polyacrylamide gel electrophoresis and electro-transferred onto polyvinylidene fluoride membranes (Immobilon P, Millipore). Membranes were incubated first with 5% non-fat dry milk in 100 mM Tris pH 7.5, 150 mM NaCl and 0.1% Tween 20 (TTBS), then with anti-PrP antibodies overnight at 4°C or 1 h at room temperature, rinsed three times with TTBS and incubated 1 h at room temperature with horseradish peroxidase-conjugated secondary antibody (diluted 1:5,000; Santa Cruz). Signals were revealed using enhanced chemiluminescence (Amersham Biosciences), and visualized by a Biorad XRS image scanner. Quantitative densitometry of protein bands was done using Quantity One software (Biorad).

### XBP1 Splicing

Total RNA was extracted using a commercial kit (SV Total RNA Isolation System; Promega, Madison, WI), according to the manufacturer's instructions. RNA samples were reverse-transcribed with MuLV Reverse Transcriptase (Applied Biosystems) by priming with oligo(dT). XBP1 mRNA was amplified with primers flanking the 26b intron (5′-GGAGTGGAGTAAGGCTGGTG and 5′-CCAGAATGCCCAAAAGGATA) and PCR products resolved on 2.5% agarose gels [17].

### Immunofluorescence Staining

Cells grown on 8-well 15-μm slides (Ibidi, Martinsried, Germany) were washed with phosphate buffered saline (PBS) and fixed for 30 min at room temperature with 4% paraformaldehyde in PBS. They were then washed with PBS, incubated with blocking solution containing 0.1% saponin, 0.5% BSA, 50 mM NH_4_Cl in PBS, and with primary and Alexa (Molecular Probes)-conjugated secondary antibodies diluted in the same solution. Cells were stained with 1 μg/ml DAPI (Sigma Aldrich) for 5 min and analyzed with an Olympus FV500 laser confocal scanning system.
